# Flow experience and city identity in the restorative environment: A conceptual model and nature-based intervention

**DOI:** 10.3389/fpubh.2022.1011890

**Published:** 2022-11-11

**Authors:** Mei Xie, Yanhui Mao, Rui Yang

**Affiliations:** ^1^School of Foreign Languages, Southwest Jiaotong University, Chengdu, China; ^2^Dipartimento di Psicologia dei Processi di Sviluppo e Socializzazione, Sapienza Università di Roma, Roma, Italy; ^3^Institute of Applied Psychology, Psychological Research and Counseling Center, Southwest Jiaotong University, Chengdu, China

**Keywords:** flow experience, optimal experience, city identity, place identity, urban identity, restorative environment, restorative experience

## Abstract

Within environmental psychology, the restorative environment is receiving increasing attention due to its favorable impact on people's mental recovery, stress reduction, and psychophysiological well-being. Flow theory, as one of the foundations of positive psychology, is a popular theoretical framework for understanding human flourishing and well-being. The restorative environment is suggested to facilitate flow experience and city identity from the perspective of positive environmental psychology. Nonetheless, systematic research investigating them all together can hardly be traced. Thus, through a preliminary review of 169 relevant studies retrieved from the data source, this work proposes a novel theoretical model in which people's interactions within the restorative environment facilitate their experience of flow and perceived city identity. Additionally, this research provides conceptual guidance for city workers to engage in nature-based intervention and leisure therapy for improved well-being. Overall, this review endeavors to contribute to developing urban workers' restoration, happiness, and well-being from both practical and theoretical perspectives.

## Introduction

Psychological stress has been identified as a significant health risk in modern societies and is, directly and indirectly, responsible for immense costs to health systems and socioeconomic well-being. Consequently, strategies for coping with psychological stress—e.g., facilitating a better working environment and promoting a healthier lifestyle—have increased interest in many research records worldwide. High levels of employee stress have been related to high levels of absenteeism and lower levels of productivity (1, 2). Workers who believe their workplaces are unsuited for their work duties are more likely to report worse well-being and poorer performance outcomes (3). Providing good work surroundings is a crucial aspect of modern office design (4). Increased utilization of urban green space during times of stress, such as the COVID-19 pandemic, has the ability to mitigate some of the stressor's detrimental consequences (5). People in the city of Shanghai in China are starting to realize and indicate their willingness to pay for green offices (6). Nonetheless, empirical studies are still needed in this field.

The “happy-productive worker” thesis states that a happy worker would perform better than an unhappy one (7). The hedonic perspective of well-being refers to a view of pleasure and the experience of positive emotions [e.g., (8)]. In contrast, the eudaimonic philosophy of well-being refers to a view of “worthwhileness” (reward) and commitment (engagement for meaning and life purpose) that is associated with the activities carried out at work (9, 10). Flow involves remaining “focused” and “engaged” in the task at hand (11). It is defined as when engaging in an activity (i.e., work), people will enter into an affective and cognitive state characterized as total absorption, full concentration, time distortion, and optimal enjoyment (12, 13). Defined as an environment that helps one recover from mental fatigue (the fatigue of directed attention), a restorative environment (14, 15) is commonly believed to be a predictor of the flow experience, a state of profound enjoyment and satisfaction elicited from daily activities (11, 14, 16, 17). Scholars have also suggested the mutually reinforcing relationships between the restorative environment and the place identity of people residing in the city (18).

Standing on the social identity approach, the self-awareness of one's membership in a place-related community, including the emotional and value-laden implications of this membership, is known as place identity (19). Place identity within environmental psychology is defined as “memories, conceptions, interpretations, ideas, and related feelings toward specific physical environments as well as types of settings” [(20), p. 60], and the “physical world socialization of the self” [(20) p. 57]. It is a facet of self-identity comparable to other identities but pertaining to people's identification with a specific geographic place (21), such as a city where people live in. Each city has its own identity, formed by images and recollections, both good and bad. As a result of much implicit evidence on flow-identity associations (21–26), it can be argued that there is an interaction between restorative environment, flow experience, and city identity. For instance, scholars have considered residential neighborhoods familiar, restorative, memorable, and beautiful environments that can significantly facilitate the flow experience (27). However, there are still no systematic studies simultaneously on these three constructs, to the best of our knowledge.

Nowadays, for those city workers and city dwellers whose a great deal of time is spent on long office hours during work, staring at the screen and immersing in diverse online virtual platforms after work (28), creating an environmentally friendly workplace and leisure space in the city that brings about restoration (29), is critical to promote more positive affective and cognitive psychological experience such as flow experience and city identity. The present work, guided by three specific goals, reviews the research on flow, city identity, and restorative environment and interprets this research within the framework of positive psychology (the flow theory—the foundations of positive psychology) and environmental psychology (attention restorative theory, ART). The first goal is to review empirical research to construct a new conceptual model relating to notions of restorative environment, psychological flow, and city identity. The second goal is to build a preliminary conceptual model based on the theoretical view of the restorative environment (14). The third goal is to identify nature-based interventions for city workers to promote their everyday flow experience and build and enhance city identity experience *via* a therapeutic process. The organization of this review maps these goals. It ends with a discussion of findings with corresponding implications for research and practice to make urban places more appealing to city workers in terms of restoration and physical activities.

## Literature review

**Figure 1:**
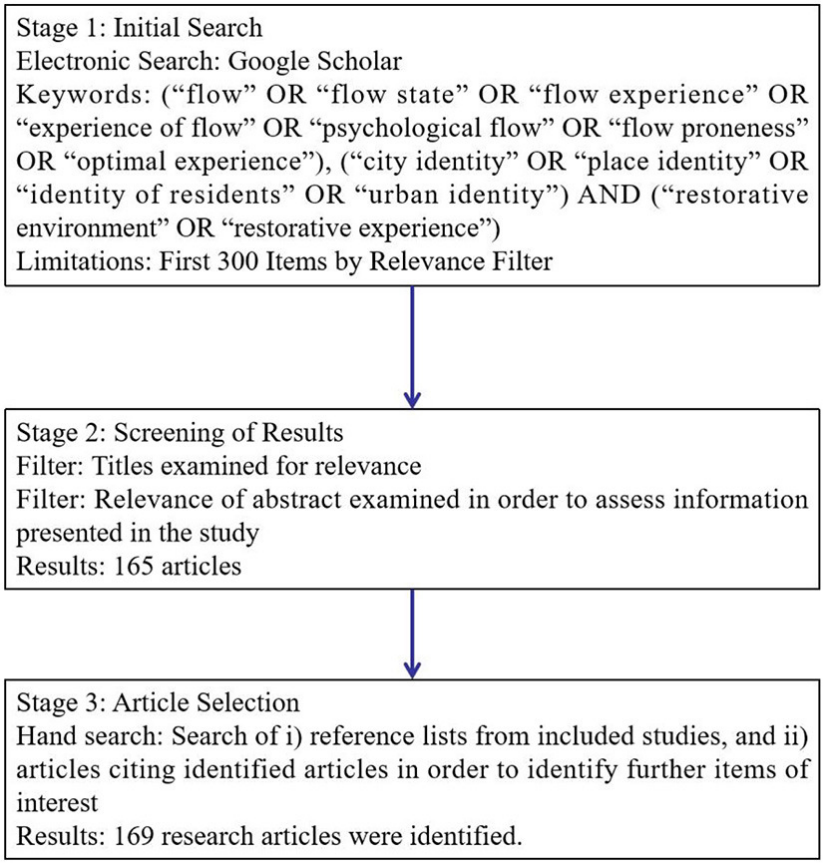
Steps for data sourcing.

A brief literature review involved the following search terms by relevance filter within the web-based search engine Google Scholar in April 2022: flow (“flow” OR “flow state” OR “flow experience” OR “experience of flow” OR “psychological flow” OR “flow proneness” OR “optimal experience”), city identity (“city identity” OR “place identity” OR “identity of residents” OR “urban identity”), AND restorative environment (“restorative environment” OR “restorative experience” OR “restorativeness”). Since the number of recorded literature simultaneously on these three constructs was small and limited, we did not restrict the type of related publications (empirical studies, articles, editorials, commentaries, opinion papers, and textbooks) to mitigate the risk of missing something significant from a study that was not appropriately cataloged within a keyword catalog such as “city identity”. However, we analyzed only the first 300 items sorted out by relevance with the following principles: (a) Articles were excluded if the abstract showed that the article did not even partially address the three variables as described above or if the construct under research was labeled as such yet addressed a different topic. If there was any doubt, the complete article was screened before deciding whether to include or exclude it; (b) They did not contribute in part to addressing the research question, or; (c) They were not available electronically or *via* other acceptable methods. The authors conducted this retrieval process twice to ensure its accuracy. We, therefore, found 165 articles (flow and city identity: 10; flow and restorative environment: 59; restorative environment and city identity: 96) among these 300 sourced items. Due to limited article space, we deleted articles with basically the replicated findings or comparably low citations after reading the abstract. There were no studies on the three pillars concurrently, yet there was still quite limited research exploring the relationship between flow and city identity. With other supporting evidence on the interventions included, a total of 169 works (e.g., 15 books, 2 conference proceedings, and 152 journal articles) were retained for review in the present work. The information sources, search terms used, and study selection procedure are outlined in Figure 1.

### Nature experience and flow

Natural surroundings are more beneficial to the psychological restoration of city dwellers than an urban environment relatively devoid of nature (30). Potential pathways connecting greenspace to health are presented in three domains, which emphasize three general functions of greenspace: reducing harm (e.g., being less exposed to air pollution, noise, and heat), restoring capacities (e.g., attention restoration and physiological stress recovery), and building capacities (e.g., promoting physical activity and enhancing social cohesion). Interrelations among the three domains also deserve further investigation (31, 32). Scholars have indicated three significant findings in terms of flow experience and the natural environment: (1) Youth indicating a preference for nature might be more active and involved in daily tasks at hand; (2) Adults value nature's restorative qualities; youngsters prefer competitive and demanding experiences and; (3) Programs should encourage, educate, train, and provide the opportunity for youth to participate in healthy nature-based activities (33–35), laying a foundation for our research on nature-based intervention for the youngsters. Indeed, when interacting with the whole world and blurring the line between self and environment, a holistic feeling of flow matters since there is neither stress nor boredom in such an environment (26, 36).

Flow experience has three prerequisites (clear goals, detailed feedback, and challenge-skills balance), three characteristics (action-awareness merging, concentration on the task at hand, sense of control), and three consequences [loss of self-consciousness, the transformation of time, autotelic experience, (37)]. Take forest experience as an example of nature experience. First, such an immersion nature experience needs prerequisites. As for “clear goals,” forest users' primary reasons seem to be a desire to escape from everyday hassle and alleviate stress (38, 39). According to the flow model, a forest experience can be classified as a challenge; to enjoy and be sensitive to a forest, one needs “skill” (i.e., training in hiking skills, knowledge about the destination landscape, and awareness of the potential dangers). Furthermore, according to Maslow's hierarchy of needs (40), the totality of a forest experience is stunning in that there is a complete concentration of attention on the forest. A person in a forest setting may be exposed to “forest stimuli” or impressed by the forest's magnificence and mystery. As for the consequences, when a person enters into a “flow” state, they may forget about the outside world (41) and, *via* communicating with nature in the forest, may achieve a condition of inner peace and tranquility similar to that experienced during a religious experience.

Additionally, some research has examined the flow concept regarding causal links with natural environments, experience levels, and related affective states (42). For instance, Tsaur et al. (43) have discovered that the transcendent aspect of high-altitude mountain climbing was a significant predictor of flow and that experiencing flow made climbers “happier”. This association is confirmed in surfing activities (44). The visitors report varied emotional assessments (i.e., awe-inspiring, peaceful, upsetting, and uninteresting) and transcendence (diminutive and deep flow) experiences in two diverse natural habitats [wild cliffs and groomed gardens, (45)]. A critical requirement for experiencing flow for these various recreational experts was the ideality and restorativeness of the natural environment, irrespective of terrestrial or aquatic. Indeed, these qualities of the flow state are not only applied to a person experiencing hiking in a mountain or forest that is not often available in daily life (46); flow can also occur in parks and the urban greenspace, which plays a role in people's everyday lives in urban areas. Maurer et al. (47) find that trees are the most significant aspect of nature, contributing to the subjective well-being and connectedness to nature of the majority of participants. Also, it has been proved that increasing employees' interaction with vegetation can enhance employee well-being and performance (48). In a word, flow can be experienced through interaction with nature, which has implications for city workers' well-being.

### Flow experience and restorative environment

The field of restorative environment research is thriving due to two major theories: Attention Restoration Theory (ART), (14) and Stress Reduction Theory (SRT) (49–51), as well as a substantial body of empirical studies. According to ART, continuous use of directed attention—actively and deliberately focusing on a task—contributes to mental fatigue, and that involuntary attention—attention that needs little to no effort—is required to alleviate directed attention fatigue. Individuals benefit from the opportunity to (1) “be away” from daily hassles and stresses, (2) encounter vast spaces and contexts (“extent”), (3) participate in activities that are “compatible” with our intrinsic motivations, and (4) critically experience stimuli that are “softly fascinating” (15). This combination of elements promotes “involuntary” or “indirect” attention while also allowing our “voluntary” or “directed” attention capabilities to repair and restore (15, 52). SRT indicates that psychological restoration may benefit affect, cognition, behaviors, and the physiological system. On the contrary, positive psychology says that people might achieve well-being and stress alleviation through what is known as flow experience, which entails people's deliberately participating in an engaging activity (11, 53), people's attention and concentration are distracted away from everyday duties and undesired ideas when they are engaged in an exciting activity (e.g., sports, yoga, writing, or socializing).

After affirming the interactive effects of attentional state with activity-setting and with social context (54), Staats (52) further describes ART and SRT theories' content, similarities, and differences. He then discusses research conducted in a variety of environmental domains, including nature (both wild and managed), the home, the workplace, museums, religious environments, hospitals, other healthcare settings, and favorite places.

As anticipated by ART, some research demonstrates people's preference for natural outdoor spaces over built-up outdoor and interior spaces such as urban settlement areas, loading docks, parking lots, and roadways (55–58). The restorative benefits of well-known cultural contexts (such as museums, industrial heritage sites, and historic city centers) that serve as tourist attractions (59) may differ from those of ordinary residential surroundings. Based on SRT and ART, evidence has shown that environmental preference has a phylogenetic basis and is intimately related to the claimed restorative benefits (60). Indeed, flow theory and SRT are expected to be connected by a significant decrease since stress and flow are embedded in an inverted U-shaped relationship (61).

Researchers have established a variety of conceptual frameworks for considering the human transformations that occur in nature, including “extraordinary experience” (62), “transcendent experience” (63), and “peak experience” (64). Extraordinary experiences include action and consciousness, attention or focus, personal integration, personal control, power awareness, joy and value, and a spontaneous letting go of process (65). Transcendent experiences are defined as those that exist beyond human observation and comprehension. They include “communion with nature that both escape from duties and the emotional relationship established by immersion in regions of unusual natural beauty” [(43) p.361]. According to Williams and Harvey (16), optimal experiences foster mental attention, immersion in the present moment, and personal growth.

Nature programs enable veterans to enjoy “optimal”, “transcendent”, or “extraordinary” experiences that incrementally demand and control skill and challenge levels (66). Grant et al. (67) also add an expanded flow theory to an ethnographic study of hobby fly fishing to demonstrate how customers' bodies, the environment, and fishing equipment contribute to restorative experiences. The experience of a VR-based restorative environment has a favorable healing impact on patients with mild-to-moderate anxiety and depression, as shown by its effects on positive and negative emotions, self-efficacy, and cognitive function (68). In a nutshell, restorative environment and flow experience are related to the individual well-being paradigm. The related intervention may be efficacious in improving attention and mood among city workers.

### Flow and city identity

Flow experience (37) has been the subject of extensive empirical research spanning more than four decades. Nonetheless, progress in understanding—beyond what Csikszentmihalyi discovered in 1975—has been minimal. To rectify this, the idea of flow must be introduced to explore how factors external to the personal influence the present-moment experience. Relational places are composed of a complex of social, material, and ecological processes operating at several scales (69), with which a person interacts and is molded (70). Human beings are exposed to various everyday activities *via* their physical and environmental surroundings. The importance of activities in defining human-environment interaction has been extensively studied in the discipline of environmental psychology (21). While attention to place-related identity is growing in environmental psychology (71), less focus has been made on developing a deeper understanding of place identity at the local level and the social nature of urban identity (26).

The discovery model of identity development postulates that adolescents' subjective identity-related experiences, such as personal expressiveness, flow, and goal-directed behavior, partially reflect these development processes (72). Bonaiuto et al. (73) demonstrate that specific indicators measuring the quality and quantity of activities associated with a particular neighborhood community can be related to neighborhood attachment. Kyle and colleagues discover that hiking activity predicts one's place identity (74), implying a cognitive relationship between the self-concept and the location of the activities (46). Bonaiuto and colleagues (36, 75, 76) have consistently found that distinct sets of activities performed by residents in different geographic locations (i.e., their neighborhood community, city center, and suburb) are related to how residents assess the environmental quality of these locations and how they ascribe belongingness to these locations (such as their neighborhood attachment and community identity).

Leisure activities contribute significantly to individual identity creation in urban industrial societies. For example, substantial studies have reviewed research on the association between teenage (positive) identity construction and leisure situations [flow, (77)]. Locations such as homes influence people's flow experience (78). Cohen (79) revisits the dialectics of escapism (flow experience), authenticity, and identity in leisure and tourist activities. Lee and Shen (80) discovered that participation in leisure activities (such as walking their dogs in urban parks) is associated with place loyalty. “An analysis of the reported experiences of people involved in various play-forms (i.e., rock-climbing, chess, dance, basketball, music composition) suggests that the qualities which make these activities enjoyable are the following: (a) a person can concentrate on a limited stimulus field, (b) in which he or she can use his or her skills to meet clear environmental demands, (c) thereby forgetting his or her own problems, and (d) his or her own separate identity, (e) at the same time obtaining a feeling of control over the environment, (f) which may result in a transcendence of ego boundaries and consequent psychic integration with personal meta systems” [(81), p 113]. Sports activities also aid career advancement by facilitating severe leisure and forming social identification (82).

Similarly, Orta et al. (83) reveal a strong link between flow experience and athletic identity. Based on the eudaimonistic identity theory (EIT), which emphasizes self-defining activities as critical for an individual's identification of their goals, values, beliefs, and interests related to the development or enhancement of one's own identity, and based on flow theory, which holds that certain salient features of an activity experience are associated with happiness and well-being, it has been shown that promoting a person's flow experience inside psychologically meaningful settings may help sustain or enhance one's place identity. Therefore, the people–place interactions support the flow-place identity link (21).

Emplaced flow considers the socio-spatial dynamics that impact a person when they strive to get engrossed in a geographically located activity and the effect of location on instantaneous sensations (84), which is a direction for future related research. Therefore, flow and city identity can be closely related, and this demands more empirical research to testify and design related interventions for city workers' enjoyable flow experience, identity building and well-being.

### City identity and restorative environment

For the most part, theory and practical findings on place identity and restorative environment have received accumulating attention in recent years, though they have developed separately. For instance, Korpela (85) proposes that place identity is a product of environmental self-regulation. Korpela and Hartig (18) examine how people value their favorite environmental locations using restorative environment theory-based criteria because a “sense of community” includes not just social relationships but also the bonds that individuals build with their natural surroundings (86). By correlating location preferences to place meaning, Kyle et al. (87) also investigate the link between place motivation and attachment. Devine-Wright and Howes (88) reveal that for people with solid place attachments, the conflict between project and place (restorative environment for inhabitants) is seen as a challenge to identity, resulting in negative attitudes and oppositional behavior. Scholars have also highlighted the importance of the relationship between landscape, place identity, and restorativeness (89). Those with an urban place-related identity (i.e., city dwellers) have viewed urban and natural settings as equivalent in terms of their ability to aid recovery from directed attention fatigue; and, on average, urban geographical locations are judged to be “rather” likely to result in restoration instead of the non-restorative outcome predicted by ART (90). Urban green space has the potential to match the effect of nature on some restoration outcomes, and environmental preferences are indicative of place identity (91). Built environments that are “attractive” are just as restorative and emotionally uplifting as natural spaces in the urban environment (92). A particular sense of place [(93), coined the term “seasides”] shows itself *via* positive and uplifting perceptions of the multisensory seaside environment; participants report a belief in the seaside as a “tonic”. The expansive views, fresh air, sea scent, and sound of crashing waves are constantly alluded to and characterized in restorative terms. Morton et al. (94) hypothesize that the restorative capacity of surroundings is governed, at least in part, by social and psychological processes associated with identity.

Besides, Bornioli et al. (95) reveal that particular interactions with place linked to personal relationships, place identity, and positive feelings of the community that resulted in improved psychological well-being. Liu et al. (96) find that landscape elements enhance individuals' attachment to new environments. Restorative impressions are favorably associated with local landscape traits, place reliance, and place identity (97). In a word, though there are quite limited studies exploring the associations between restorative environment and city identity, place identity plays a crucial role in mediating the naturalness-wellbeing link (98). Based on this, a more nuanced understanding of the potential relationship between city identity and a restorative environment is necessary, since a diversity of (natural and urban) locations may be restorative and related to city workers' identity (29).

### Flow, city identity, and restorative environment

Research records on the relationships among flow, city identity, and restorative environment cannot be traced for the time being, but much implicit sparking evidence can be traced in extant works, such as the following: Tsaur et al. (99) identify 6 factors to quantify the fit between recreationists and their environment based on Attention Restoration Theory and Affordance Theory: natural resources, interpersonal opportunities, environmental functions, facilities, activity knowledge/skills, and operation/management. It is *via* flow experiences that we strengthen our place attachment, choose our favorite places, and generate intrinsic motivation to enter certain places but not others (100). A growing body of research shows that adventurous nature sports can promote diverse hedonic and eudaimonic aspects of well-being, including: (a) facilitating feelings of connection to nature; (b) fostering physical and mental benefits associated with physical activity, (c) providing opportunities to overcome challenges and have optimal experiences; (d) increasing positive psychological outcomes such as positive affect, self-efficacy, and resilience; (e) restoring cognitive resources; (f) providing opportunities to experience self-determination (e.g., *via* psychological need fulfillment and intrinsic value orientations); and (g) promoting social connectedness (101).

Williams and Harvey (16) divide forest experiences into six distinct categories, one of which—deep flow—can be characterized as transcendent experience. The deep flow experience is distinct. It includes a high degree of compatibility and a moderate degree of novelty and is thus likely to be reported as relaxing or eliciting a sense of belonging.

Rosenbaum and Massiah (102) demonstrate how consumer reactions to social, symbolic, and natural stimuli often serve as the catalyst for solid person-place attachments and how a servicescape's inherently restorative feature may alleviate mental fatigue and enhance customer health and well-being.

Bell et al. (103) examine how symbolic, achievement-oriented, immersive, and social experiences contribute to participants' sense of well-being in their local coastal regions, using a unique adaption of the therapeutic landscape concept.

Morton et al. (94) establish that exposure to nature vs. urban images enhances cognitive ability and good motivational states, and salient identities influence how individuals react to natural environments. In a nutshell, they argue that the environment's restorative capacity is partly influenced by social and psychological processes associated with identity, and a more nuanced understanding of the potential psychological benefits of exposure to nature is necessary and that a diversity of environments (natural and urban) may be restorative.

Lee et al. (104) investigate the mechanisms behind the therapeutic benefits of the urban forest on middle-aged women through their participation in an urban forest therapy program. Following these women's acquisition of information about the forest and emotional connection with one another, their mental attitudes shift, allowing them to identify with nature and think about their own lives. They are then able to cultivate coping mechanisms that eventually result in self-healing. This work demonstrates how participants self-heal through interactions with nature, guides, and other group members.

Giusti and Samuelsson (105) propose that when environmental attitudes (including identification with nature) and natural environments interact statistically, almost all restorative experiences (including feeling immersed in the place—one core element of flow) are predicted more accurately than when these factors are independent predictors, that is, there is synergistic compatibility between environmental attitudes and healthy ecosystems that triggers restorative processes.

Most importantly, Peng et al. (36) find that community identity mediates the path from flow experience and green space to life satisfaction, indicating the importance of subjective enjoyable flow and objective green areas in reshaping community residents' well-being in life-challenging environments (i.e., COVID-19).

**Figure 2:**
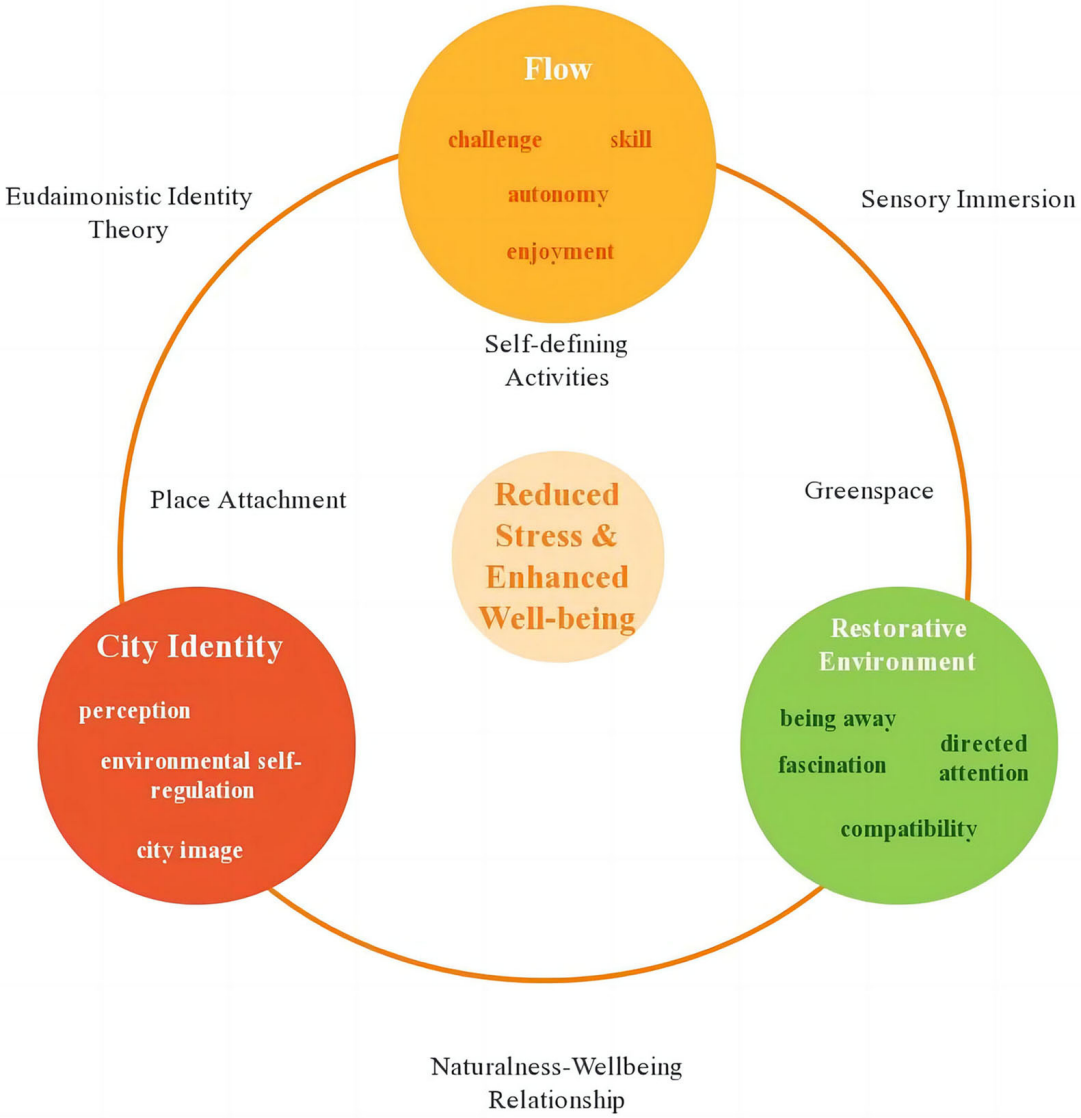
The proposed conceptual model among flow, city identity, and restorative environment.

From the above discussion, we propose that the three variables are implicated in reciprocal feedback loops, as shown in Figure 2.

## Practical intervention for city workers

In recent years, it has become more apparent that urban planning and environmental design influence mental health and well-being. According to the “happy-productive worker hypothesis”, people who are more content with their jobs are also more productive and engaged. Promoting workplace well-being and reducing work-related stress may have far-reaching repercussions, not just for knowledge-sector employees but also for the productivity of organizations, and employers have increasingly recognized this. Building on a long tradition of urban greenspace provision as a public good supporting population health [e.g., by the urban parks and garden cities movements, (106)], much recent research examining the relationship between the physical environment and health has focused on the role of green space (26, 36). By examining the links between restorative environment, flow experience, and city identity, this paper demonstrates how workplaces may be structured to accommodate these aspects. These results have implications for the planning and design of urban business sites and the development of interventions to increase employee well-being.

### Creating restorative environment

Although numerous studies have indicated that the scenic view of nature is restorative, many city workers may not have ready access to natural settings in modern society. Fieldhouse (107) finds that some characteristics of the plant-human connection facilitate people's engagement with their environment, promoting their health, functional level, and subjective well-being. Due to the allotment's embeddedness within communities, it has enormous promise as a medium for occupational therapy and a method for social integration. The evidence for environmental restoration support is divided into four categories: residential contexts, work and school settings, care settings, and other settings. We concentrate only on workplace interventions for young city workers. With the importance of nature in the workplace (48), there is a wealth of studies demonstrating a clear correlation between workers' well-being and greenspace (108–110) as well as landscapes (111). For instance, Bringslimark et al. (112) discovered that employees in windowless offices were more likely to bring plants to work or decorate their office space with a photograph of nature than those with a window view, which can be viewed as a compensatory strategy for the lack of access to the outdoors provided by windows (e.g., restoration for those in need of it). Additionally, Boubekri et al. (113) establish that workplace windows (114, 115) and daylight exposure are predictors of job satisfaction and overall health for office employees. Wang et al. (116) find that all the seven different types of forest resting environments in a virtual reality video can produce stress relief effects to some extent.

Both open space utilization and views of specific vegetation types, such as trees, lawns, shrubs, and blooming plants, are positively and independently linked with employees' self-reported well-being levels (117). For example, Zurawik (118) indicates that indoor plants are connected with a decrease in office employees' sick absence frequency and increased productivity, owing to the assistance offered by plants for healing over time. Inadequate indoor air quality adds to employee health and well-being degradation, further reducing productivity; the design solution should, therefore, incorporate aspects that promote cooperation and teamwork among workers and adaptable and ergonomic furniture to boost productivity. Environmentally friendly materials and furnishings should also be chosen to safeguard staff health, well-being, and global ecosystems (119). White et al. (120) report that spending at least 120 min a week in nature is associated with good health and well-being, so stakeholders should pay attention to such a “threshold” and actively develop possible weekly nature exposure guidelines for their employees.

Gritzka et al. (121) conducted a systematic review of the scientific evidence on the usefulness of nature-based interventions in promoting mental health and well-being among workers in real work contexts.

There is considerable consensus about the beneficial impacts of exposure to nature. For instance, natural elements decorated with green in the office help with stress reduction, with red, yellow, and orange stimulating positive emotions (122). Adding one to three green plants to the office table facilitates employees' productivity, focused attention, and stress reduction, hence promoting well-being (123). Designing offices with outdoor natural window views promotes work productivity (124), and equipping green areas in the community promotes dwellers' sense of belonging and attachment (76). As a result, nature-based interventions in the workplace have been advocated as a cost-effective strategy for promoting employee health (121). Research on the effect of green space on employee mental health and well-being is worthy to assist in the design of workplaces. Thus, we suggest that all stakeholders engage in creating a restorative environment by, for instance, developing a workplace equipped with a small green space (indoors: e.g., indoor garden, or outdoors near the workplace, 108), designing an employee break room decorated with nature elements (125), and exploiting virtual reality nature simulations [such as playing a video with natural sceneries, (126)], and so on and so forth.

### Flow interventions for city workers

Although several flow-based interventions have been developed and evaluated (127), most have targeted general workers, not those who may not have ready access to natural settings. According to Wöran and Arnberger (17), leisure specialization and restorative surroundings are linked to flow experience. It is widely proven that employees' leisure activities are related to recovery experiences, job performance, and subjective well-being (128–130). According to Caldwell (131), leisure activities (e.g., learning how to generate a flow-like experience) may contribute to physical, social, emotional, and cognitive health through prevention, coping (adjustment, remediation, diversion), and transcendence. Flow is essential for adherence to regular workplace physical activity, such as football and Zumba (132). Also, the importance of mindfulness training in the workplace cannot be overstated (133, 134).

Various factors can contribute to frontline employees' job flow experience, such as personal factors, job characteristics, and leadership (135, 136). Even in a dynamic workplace environment, the rhythms of attentional states remain context and time-dependent (137). Focus is most significant in the mid-afternoon, while boredom is at its peak in the early afternoon. People are happiest while performing routine tasks and most agitated when performing focused tasks. Mondays are the most boring day of the week, but they are also the most concentrated (138). While flow-like experience is not exclusive to leisure episodes, the more robust flow experience is more likely to occur during freely selected activities that produce intrinsic interest for the receiver (139). Plester and Hutchison (140) examined the relationship between fun and workplace engagement using three distinct types of workplace fun: managed, organic, and task fun. They discovered that particular workplace fun provides individual employees with a refreshing break, resulting in positive affect and increased workplace engagement and task engagement.

Martens (141) emphasized the value of setting aside time and space for relaxation or taking a stroll after intense work to relieve some of the stress associated with sitting and working (e.g., writing, designing, etc.,) in one location, and justified the inclusion of gaming rooms, relaxation lounges, and greenspace in or near workplaces. Concentrated work is much easier when surrounded by nature and without distractions. Suh et al. (142) highlight the central role of the aesthetic experience on gamification in the workplace.

Through meaningful occupations in a restorative environment, nature-based rehabilitation changes everyday occupations' perceived values [including self-rewarding value—flow, (143)]. Taken together, we remind all stakeholders to realize optimal work environments and nature-based rehabilitation by considering implementing some flow interventions through goal setting (127) and deep acting (144).

### City identity interventions for city workers

To our best knowledge, there are so far no city-identity-based interventions focused on supporting city workers to cope with work stress. We try to propose some potential strategies inspired by place identity intervention. Research on community attachment reveals that integration into the local region significantly predicts connection to place. Local social interactions—particularly with friends, relatives, organizational affiliations, and local shopping—are the most persistent and substantial sources of emotional attachment to local locales (145). Place identity is highly correlated with neighborhood satisfaction (146). Second, long-term residency leads to the development of place identity; the length of stay strengthens local social bonds (147). It also offers a chronological background for imbuing a place with personal significance. The Hukou system continues to be a barrier to Chinese migrant workers; despite their economic, social/cultural, and identity absorption into urban life over time, human capital plays a critical role in migrants' economic and identity integration (148). Workers' intention to leave their current location is connected to their sense of place identity and fairness judgments (149).

The place identity is built *via* people's cognitive identification and satisfaction with the place, as well as their affective enjoyment and sense of security when interacting with the place. Place identity is a dynamic and dialectic process, and the memory of a residential place has a positive relationship with place identity and place attachment (150). Unsuitable interventions will erode both the physical environment and the sensation of attachment rooted in people's attachment (151). Thus, planners must incorporate visions of place identity not only from politicians and economic interests but also from residents and other members of civil society because the identities ascribed to a place by various stakeholders are contested and may play a role in social conflicts (152).

City identity may develop through individual, group, or cultural processes. More recently, our research team has found that a better-perceived quality of the residential place (i.e., neighborhood community) is associated with a higher level of community identity (26). Community involvement, including social bonds, community participation, and community empowerment, is the most critical factor in determining the degree of community attachment (153). Therefore, governments, companies, and other stakeholders should take measures to enhance the city identity of young frontline workers by giving value to the city through place memory, including increasing welfare entitlements (154), community involvement, as well as justice perceptions (149), thereby increasing their belongingness and reducing their work stress.

### Combined interventions

Similarly, there are to date almost no interventions simultaneously on flow, city identity, and restorative environment developed to address workplace stress. We try to propose some practical interventions in the time and effort they demand of all parties and the resources necessary to implement them, which is also the most significant contribution of the present study.

Valera and Vidal (155) discussed several theoretical advancements in environmental psychology and made a case for their inclusion in a Positive Environmental Psychology agenda. On the one hand—space as a source of well-being—they examine the aesthetic quality of landscapes, the restorative potential of surroundings, and the evolution of place identity and place attachment. On the other hand—space as a context for positive experiences—they propose some reflections on urban placemaking processes, such as the tradition of Placemaking, Community Participation, and Planning, or the Socially Restorative Urbanism movement, which aims to restore social well-being and a sense of belonging to urban environments.

Uzzell et al. (156) examined the effects of social cohesion, residential satisfaction, and place identification on place-related social identity and its consequential impact on attitudes to environmental sustainability, suggesting a significant relationship between identity and sustainable behavior that is suggestive for future research, i.e., companies should create nature-based suitable accommodation for workers. Free-time physical activity in natural surroundings after work is a potential strategy for enhancing employee vitality across time (157). Employees are expected to have quality restorative break time after work. Time-flexible work policies can reduce stress, improve health, and save money (158), which should be encouraged. Job control and work-life balance practices moderate frontline employees' stress (159). Employee recreation welfare is quite essential (160). Employee recreation/fitness programs in the rest space and worksite lifestyle change programs should be encouraged (161). Psychological commitment mediates the influence of leisure involvement on the flow experience during hiking activity, so some training programs in the wild to develop employees' organizational commitment are significant (162). High technology should also be introduced, such as a portable biofeedback device (163). Live plants and window views of green spaces affect employee perceptions of job satisfaction (164), so qualified leaders should be selected for young city workers to collaborate with the faculty of forestry, the institute of mental health, and the botanical garden in the local place to improve employees' health. Also, a competent support crew is expected to adopt a biophilic design and increase (indoor) green plants.

Increased conversation between flow and therapeutic environments elucidates aspects that assist therapeutic experiences, therefore, defining how person-place interactions might be created to promote well-being. Pitt (84) establishes flow in community gardens by examining how spatial factors affect the therapeutic activity and how social relationships affect the possibility of discharge. Other therapeutic activities might be explored similarly to ascertain other socio-spatial variables that facilitate or obstruct flow experience. Further study might be beneficial in determining if therapeutic hobbies such as gardening have a decreasing return when repeated or routine and whether brief getaways have long-term effects on wellbeing.

Hawkins et al. (66) argue that nature-based interventions typically incorporate foundational elements from Flow Theory (e.g., challenge matching skills) and ART, as well as other theoretical qualities such as buffering effects; a sense of remoteness, escape, and awe; and an attachment of a sense of meaning to the place itself. At the heart of their methodology are nature-based therapies motivated by the individual's internal and external strengths and current assets (e.g., character strengths and skillsets). These advantages distinguish nature-based treatments from conventional medical model interventions. A recreation therapist aware of these talents and abilities before the intervention *via* an assessment process will call on them during the intervention to facilitate a positive experience. Finally, the model's top layer depicts the results of nature-based therapy, which include enhancements in social, cognitive, and spiritual domains and outcomes related to identity, purpose, and overall healing from life issues.

Cole et al. (165) specifically examine putative connections between green building design principles and psychological processes of place attachment (i.e., affect, identity, and dependence) in non-residential buildings and reveal four critical green design strategies for promoting place attachment: (1) biophilic design and opportunities for connection to nature, (2) visible environmentalism, (3) opportunities for pro-environmental behaviors and (4) an indoor environmental quality that promotes physiological comfort.

Greenspace in urban areas is a vital health resource, facilitating physical activity, promoting social interaction, and restoring exhausted psychological resources, all of which can positively affect physical and mental health (26, 166–168), thus all relevant parties and stakeholders should increase the greenness and improve the environment surrounding workplaces.

**Figure 3:**
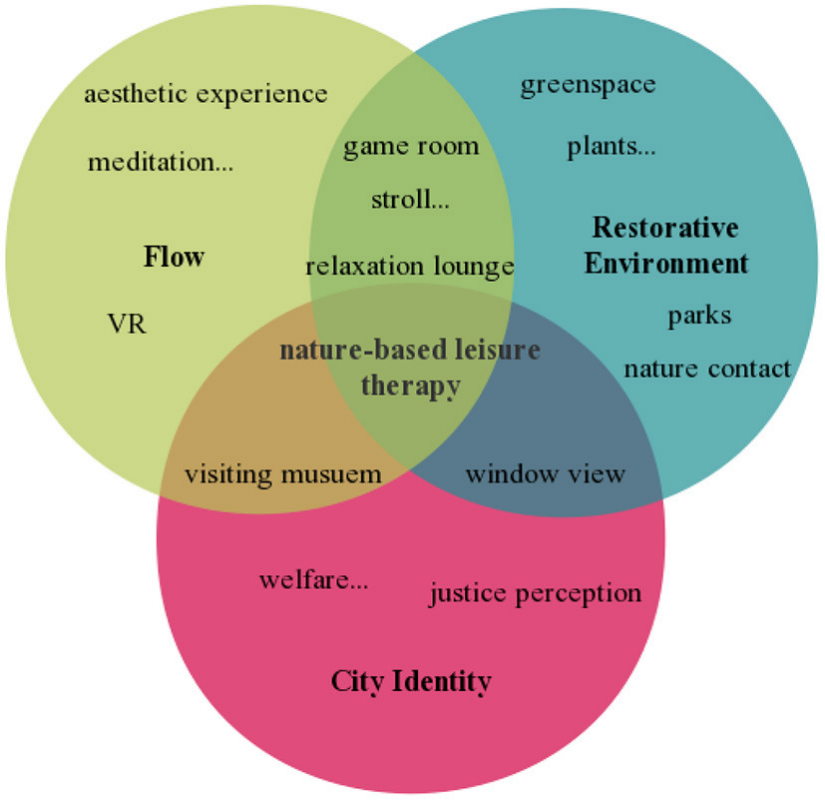
The proposed intervention model synthesizing flow, city identity, and restorative environment.

In a nutshell, managing job stress requires all relevant parties and stakeholders' cooperation to jointly create sustainable workplaces, including employee self-regulation, human resource management partnership, and compassionate city planners (169, 170). And the overlapping relationship between the interventions within each of the three pillars is shown in Figure 3.

## Discussion

Standing on the theoretical underpinnings (i.e., eudaiministic identity theory, attention restoration theory, and stress reduction theory) within positive environmental psychology, this review proposed a novel conceptual framework suggesting that a restorative environment could facilitate city workers' daily flow experience and city identity. We have explored the possibility of combining the three constructs of flow, city identity, and restorative environment to propose interventions in today's stressful workplace. The conceptual model guiding this review posits that exposure to natural environments predicts positive change in people's positive affective and cognitive experiences such as flow, which impacts their attitudes, belongingness, perceived values and norms, influencing their working city identities and behaviors. We believe that flow, city identity, and restorative environment are among the most significant topics in Positive Environmental Psychology literature, and we wish such a review helps all stakeholders recognize the full potential of flow for human flourishing and well-being at the workplace, the positive psychophysiological impact of restorative environment on city workers' health. To this end, we wish to bridge people and places (i.e., cities) *via* positive environmental psychology for a healthier life and a better working environment with which they are strongly identified.

### Study limitations

We identify the following points as defining psychological research on “city identity”: heterogeneity of terms and their spatial extension, divergent theoretical foundations and fragmented formulations, a lack of appropriate measuring instruments, and a scarcity of empirical work. This, combined with the small number of studies simultaneously on the three constructs, makes it challenging to identify a pattern and should be considered when interpreting the results. The fundamental affective and cognitive flow is likewise perceived differently by various instruments. Thus, the continued development and use of empirically validated measures is a critical next step for research on the dynamic relationship between flow, city identity, and restorative environment. Another issue is that although there were significant overlaps between adopting flow and healthy environments, many studies only look at the restorative environment (141). In a word, we try to put forward a conceptual model within which the three constructs studied separately are simultaneously discussed here.

This review represents the first creative synthesis of the specific mechanisms of the restorative environment through which people's flow experience is associated with their city identity, evidencing several theoretically coherent patterns. It should be noted, of course, that studies on a particular pathway or process were sometimes too few to draw definitive conclusions. This review should be considered preliminary, and more work is needed to understand further and verify these clear pathways. Nevertheless, this review can help orient researchers to the paths by which a restorative environment can contribute to city workers' reduced stress, increased flow, and stronger city identity.

One limitation of the present review is that the literature search is done from Google Scholar, only taking highly relevant articles (the first 300 items) into account. For this reason, more complex and potential relationships may be discovered, and comprehensive results may be obtained by examining as many databases as possible in future work. Another limitation of the present review is that there are various populations in the modern city, such as teachers vs. students, elderly residents vs. the young, and so on and so forth. However, in this study, we only included and explored some feasible interventions for city workers, therefore, further studies to qualitatively and quantitatively explore other population groups might advance our understanding of the influential factors for the development of sustainable cities, park cities, and happy cities.

### Implications

The current review advances related theories by identifying pathways that explain how people's flow experience and city identity are associated with the restorative environment and examining the interventions that may contribute to city workers' subjective well-being. This review makes a timely, theoretical contribution to the study of the restorative environment. Most scholarly attention is given to direct relations between a restorative environment and people's better health, and so little is known regarding the explanatory processes that account for these associations. The present review addresses this need by sensitizing researchers to the social-cognitive mechanisms through which a restorative environment might influence people's optimal enjoyment and identity and make it more or less effective over these social-cognitive mechanisms. The practical implications are that urban planners and company managers who act in turbulent environments may include new relationships between relevant and current variables in their city/workplace design, such as those associated with the city identity and restorative environment. These new approaches may help business executives use interventions that approach more of the business reality, making their workplace more harmonious and enjoyable.

## Conclusion

Through the lens of environmental psychology and positive psychology *via* restorative environment and positive affective and cognitive experience of flow, the present work attempts to develop a novel conceptual model by emphasizing that engaging in the natural restorative environment is inducive to the universal human experience of enjoyable flow and enhanced city identity, as well as their stress recovery and well-being. To date, no systematic research has contemporarily addressed the underlying mechanisms among these three constructs. Therefore, this review represents a starting point and hopes to encourage further empirical research on how to assist city workers' well-being and develop sustainable cities through brief and practical nature-based interventions for facilitating flow and city identity. We hope there could be further cues for a positive environmental psychology agenda to promote city workers' human flourishing and well-being, build their city identity, and facilitate the public health of the cities.

## Author contributions

MX: conceptualization and writing—original draft. YM: conceptualization, writing—revision and editing, and funding acquisition. RY: conceptualization and writing—revision and editing. All authors have read and agreed to the published version of this manuscript.

## Funding

This research was funded by the National Natural Science Foundation of China (Grant Nos. 71801180 and 72271205), the 2022 Southwest Jiaotong University Key Project of Degree and Postgraduate Education and Teaching Reform (YJG5-2022-Z019), and the Applied Psychology Research Center of Sichuan Province (CSXL-22101).

## Conflict of interest

The authors declare that the research was conducted in the absence of any commercial or financial relationships that could be construed as a potential conflict of interest.

## Publisher's note

All claims expressed in this article are solely those of the authors and do not necessarily represent those of their affiliated organizations, or those of the publisher, the editors and the reviewers. Any product that may be evaluated in this article, or claim that may be made by its manufacturer, is not guaranteed or endorsed by the publisher.

## References

[1] Brunner B Igic I Keller AC Wieser S. Who gains the most from improving working conditions? Health-related absenteeism and presenteeism due to stress at work. Eur J Health Econ. (2019) 20:1165–80. 10.1007/s10198-019-01084-9 31309366 PMC6803571

[2] Reb J Chaturvedi S Narayanan J Kudesia RS. Leader mindfulness and employee performance: a sequential mediation model of LMX quality, interpersonal justice, and employee stress. J Bus Ethics. (2019) 160:745–63. 10.1007/s10551-018-3927-x

[3] De Clercq S Fontaine JR Anseel F. In search of a comprehensive value model for assessing supplementary person—organization fit. J Psychol. (2008) 142:277–302. 10.3200/JRLP.142.3.277-302 18589938

[4] Wohlers C Hartner-Tiefenthaler M Hertel G. The relation between activity-based work environments and office workers' job attitudes and vitality. Environ Behav. (2019) 51:167–98. 10.1177/0013916517738078

[5] Berdejo-Espinola V Suárez-Castro AF Amano T Fielding KS Oh RRY Fuller RA. Urban green space use during a time of stress: a case study during the COVID-19 pandemic in Brisbane, Australia. People Nat. (2021) 3:597–609. 10.1002/pan3.10218 34151197 PMC8207087

[6] Zhang Y Yin H. Willingness to pay for green office: evidence from Shanghai. Environ Sci Pollut Res. (2022) 1–12. 10.1007/s11356-022-21555-6 35779220

[7] Judge TA Thoresen CJ Bono JE Patton GK. The job satisfaction–job performance relationship: a qualitative and quantitative review. Psychol Bull. (2001) 127:376. 10.1037/0033-2909.127.3.376 11393302

[8] Diener E. Subjective well-being: the science of happiness and a proposal for a national index. Am Psychol. (2000) 55:34–43. 10.1037/0003-066X.55.1.34 11392863

[9] Cui P Mao Y Shen Y Ma J. Moral identity and subjective well-being: the mediating role of identity commitment quality. Int J Environ Res Public Health. (2021) 18:9795. 10.3390/ijerph18189795 34574719 PMC8465947

[10] White MP Dolan P. Accounting for the richness of daily activities. Psychol Sci. (2009) 20:1000–8. 10.1111/j.1467-9280.2009.02392.x 19549079

[11] Csikszentmihalyi M. Flow: The Psychology of Optimal Experience. New York, NY: Harper Perennial. (1990).

[12] Csikszentmihalyi M. The flow experience and its significance for human psychology. In: eds Csikszentmihályi M, Csikszentmihályi IS. Optimal Experience: Psychological Studies of Flow inConsciousness. Cambridge: Cambridge University Press (1988). p. 15–35.

[13] Csikszentmihalyi M LeFevre J. Optimal experience in work and leisure. J Pers Soc Psychol. (1989) 56:815. 10.1037/0022-3514.56.5.815 2724069

[14] Kaplan R Kaplan S. The Experience of Nature: A Psychological Perspective. Cambridge: Cambridge University press (1989).

[15] Kaplan S. The restorative benefits of nature: toward an integrative framework. J Environ Psychol. (1995) 15:169–82. 10.1016/0272-4944(95)90001-2

[16] Williams K Harvey D. Transcendent experience in forest environments. J Environ Psychol. (2001) 21:249–60. 10.1006/jevp.2001.0204

[17] Wöran B Arnberger A. Exploring relationships between recreation specialization, restorative environments and mountain hikers' flow experience. Leisure Sci. (2012) 34:95–114. 10.1080/01490400.2012.652502

[18] Korpela K Hartig T. Restorative qualities of favorite places. J Environ Psychol. (1996) 16:221–33. 10.1006/jevp.1996.001829250016

[19] Tajfel H. Differentiation Between Social Groups: Studies in the Social Psychology of Intergroup Relations. London: Academic Press (1978).

[20] Proshansky HM Fabian AK Kaminoff R. Place identity: physical world socialization of the self (1983). J Environ Psychol. (2014) 3:57–83. 10.1016/S0272-4944(83)80021-8

[21] Bonaiuto M Mao Y Roberts S Psalti A Ariccio S Ganucci Cancellieri U. Optimal experience and personal growth: flow and the consolidation of place identity. Front Psychol. (2016) 7:1654. 10.3389/fpsyg.2016.01654 27872600 PMC5097910

[22] Mao Y Roberts S Bonaiuto M. Optimal experience and optimal identity: a multinational examination at the personal identity level. In: Flow Experience: Empirical Research and Applications. Switzerland: Springer (2016). p. 289–308.

[23] Mao Y Roberts S Pagliaro S Csikszentmihalyi M Bonaiuto M. Optimal experience and optimal identity: a multinational study of the associations between flow and social identity. Front Psychol. (2016) 7:67. 10.3389/fpsyg.2016.00067 26924995 PMC4760053

[24] Zhang SN Li YQ Liu CH Ruan WQ. How does authenticity enhance flow experience through perceived value and involvement: the moderating roles of innovation and cultural identity. J Travel Tour Mark. (2019) 36:710–28. 10.1080/10548408.2019.1625846

[25] Mao Y Lai Y Luo Y Liu S Du Y Zhou J. Apple or Huawei: understanding flow, brand image, brand identity, brand personality and purchase intention of smartphone. Sustainability. (2020) 12:3391. 10.3390/su12083391

[26] Mao Y Peng C Liang Y Yuan G Ma J Bonaiuto M. The relationship between Perceived Residential Environment Quality (PREQ) and community identity: flow and social capital as mediators. Soc Indic Res. (2022) 163:771–97. 10.1007/s11205-022-02915-8 35431400 PMC8994697

[27] Quayle M van der Lieck TCD. Growing community: a case for hybrid landscapes. Landsc Urban Plan. (1997) 39:99–107. 10.1016/S0169-2046(97)00048-0

[28] Yee N. The labor of fun: How video games blur the boundaries of work and play. Games Culture. (2006) 1:68–71. 10.1177/1555412005281819

[29] Hartig T Kahn Jr PH. Living in cities, naturally. Science. (2016) 352:938–40. 10.1126/science.aaf3759 27199417

[30] Van den Berg AE Hartig T Staats H. Preference for nature in urbanized societies: Stress, restoration, and the pursuit of sustainability. J Soc Issues. (2007) 63:79–96. 10.1111/j.1540-4560.2007.00497.x

[31] Hartig T Mitchell R De Vries S Frumkin H. Nature and health. Annu Rev Public Health. (2014) 35:207–28. 10.1146/annurev-publhealth-032013-182443 24387090

[32] Markevych I Schoierer J Hartig T Chudnovsky A Hystad P Dzhambov AM. Exploring pathways linking greenspace to health: theoretical and methodological guidance. Environ Res. (2017) 158:301–17. 10.1016/j.envres.2017.06.028 28672128

[33] Milligan C Bingley A. Restorative places or scary spaces? The impact of woodland on the mental well-being of young adults. Health Place. (2007) 13:799–811. 10.1016/j.healthplace.2007.01.005 17383927

[34] Sklar SL Anderson SC Autry CE. Positive youth development: a wilderness intervention. Ther Recreat J. (2007) 41:223.

[35] Flett RM Moore RW Pfeiffer KA Belonga J Navarre J. Connecting children and family with nature-based physical activity. Am J Health Educ. (2010) 41:292–300. 10.1080/19325037.2010.10599156

[36] Peng C Yuan G Mao Y Wang X Ma J Bonaiuto M. Expanding social, psychological, and physical indicators of urbanites' life satisfaction toward residential community: a structural equation modeling analysis. Int J Environ Res Public Health. (2021) 18:4. 10.3390/ijerph18010004 33374936 PMC7792594

[37] Csikszentmihalyi M. Beyond Boredom and Anxiety: The Experience of Play in Work and Games. San Francisco, CA: Jossey-Bass (1975).

[38] Shin WS. The influence of forest view through a window on job satisfaction and job stress. Scand J For Res. (2007) 22:248–53. 10.1080/02827580701262733

[39] Shin WS Yeoun PS Yoo RW Shin CS. Forest experience and psychological health benefits: the state of the art and future prospect in Korea. Environ Health Prev Med. (2010) 15:38–47. 10.1007/s12199-009-0114-9 19844774 PMC2793345

[40] Maslow AH. Motivation and Personality. New York, NY: Harper and Row (1970).

[41] Scott NR. Toward a psychology of wilderness experience. Nat Resources J. (1974) 14:231.

[42] Kim M Thapa B. Perceived value and flow experience: Application in a nature-based tourism context. J Dest Mark Manag. (2018) 8:373–84. 10.1016/j.jdmm.2017.08.002

[43] Tsaur SH Yen CH Hsiao SL. Transcendent experience, flow and happiness for mountain climbers. Int J Tour Res. (2013) 15:360–74. 10.1002/jtr.1881

[44] Cheng TM Lu CC. The causal relationships among recreational involvement, flow experience, and well-being for surfing activities. Asia Pacific J Tour Res. (2015) 20:1486–504. 10.1080/10941665.2014.999099

[45] Davis N Gatersleben B. Transcendent experiences in wild and manicured settings: The influence of the trait “connectedness to nature”. Ecopsychology. (2013) 5:92–102. 10.1089/eco.2013.0016

[46] Hernández B Hidalgo MC Salazar-Laplace ME Hess S. Place attachment and place identity in natives and non-natives. J Environ Psychol. (2007) 27:310–9. 10.1016/j.jenvp.2007.06.003

[47] Maurer M Zaval L Orlove B Moraga V Culligan P. More than nature: Linkages between well-being and greenspace influenced by a combination of elements of nature and non-nature in a New York City urban park. Urban For Urban Green. (2021) 61:127081. 10.1016/j.ufug.2021.127081

[48] Kaplan R. The role of nature in the context of the workplace. Landsc Urban Plan. (1993) 26:193–201. 10.1016/0169-2046(93)90016-7

[49] Ulrich RS. Aesthetic and affective response to natural environment. In:Altman I Wohlwill JF, editors. Human Behavior and Environment. Behavior and the Natural Environment. New York, NY: Plenum Press Vol. 6 (1983). p. 85–125.

[50] Ulrich RS. View through a window may influence recovery from surgery. Science. (1984) 224:420–1. 10.1126/science.6143402 6143402

[51] Ulrich RS Simons RF Losito BD Fiorito E Miles MA Zelson M. Stress recovery during exposure to natural and urban environments. J Environ Psychol. (1991) 11:201–30. 10.1016/S0272-4944(05)80184-7

[52] Staats H. Restorative Environments. New York, NY: Oxford University Press (2012).

[53] Seligman MEP Peterson C. Positive clinical psychology. In:Aspinwall LG Staudinger UM, editors. A Psychology of Human Strengths: Fundamental Questions and Future Directions for a Positive Psychology. Washington, DC: APA Books (2004).

[54] Staats H Van Gemerden E Hartig T. Preference for restorative situations: interactive effects of attentional state, activity-in-environment, and social context. Leisure Sci. (2010) 32:401–17. 10.1080/01490400.2010.510990

[55] Hartig T Korpela K Evans GW Gärling T. A measure of restorative quality in environments. Scand Hous Plan Res. (1997) 14:175–94. 10.1080/02815739708730435

[56] Berto R Massaccesi S Pasini M. Do eye movements measured across high and low fascination photographs differ? Addressing Kaplan's fascination hypothesis. J Environ Psychol. (2008) 28:185–91. 10.1016/j.jenvp.2007.11.004

[57] Felsten G. Where to take a study break on the college campus: an attention restoration theory perspective. J Environ Psychol. (2009) 29:160–7. 10.1016/j.jenvp.2008.11.006

[58] Yang LC Ao YB Ke JT Lu Y Liang Y. To walk or not to walk? Examining non-linear effects of streetscape greenery on walking propensity of older adults. J Transp Geogr. (2021) 94:103099. 10.1016/j.jtrangeo.2021.103099

[59] Packer J Bond N. Museums as restorative environments. Curator Museum J. (2010) 53:421–36. 10.1111/j.2151-6952.2010.00044.x

[60] Hartig T Staats H. The need for psychological restoration as a determinant of environmental preferences. J Environ Psychol. (2006) 26:215–26. 10.1016/j.jenvp.2006.07.007

[61] Peifer C Schulz A Schächinger H Baumann N Antoni CH. The relation of flow-experience and physiological arousal under stress—can u shape it? J Exp Soc Psychol. (2014) 53:62–9. 10.1016/j.jesp.2014.01.009

[62] Abrahams RD. Ordinary and extraordinary experience. In: Tv W, Be M. The Anthropology of Experience. Urbana: University of Illinois Press (1986). p. 45.

[63] Watson D. A Dictionary of Mind and Spirit. New York, NY: Avon Books (1991).

[64] McDonald MG Wearing S Ponting J. The nature of peak experience in wilderness. Hum Psychol. (2009) 37:370–85. 10.1080/08873260701828912

[65] Arnould EJ Price LL. River magic: extraordinary experience and the extended service encounter. J Consum Res. (1993) 20:24–45. 10.1086/209331

[66] Hawkins BL Townsend JA Garst BA. Nature-based recreational therapy for military service members: a strengths approach. Ther Recreat J. (2016) 50:55. 10.18666/TRJ-2016-V50-I1-6793

[67] Grant A Shankar A Canniford R. Extending flow: how place, materials, and body create restorative consumption in nature. ACR N Am Adv. (2020).

[68] Li H Dong W Wang Z Chen N Wu J Wang G. Effect of a virtual reality-based restorative environment on the emotional and cognitive recovery of individuals with mild-to-moderate anxiety and depression. Int J Environ Res Public Health. (2021) 18:9053. 10.3390/ijerph18179053 34501643 PMC8430968

[69] Massey D. For Space. London: Routledge (2005).

[70] Conradson D. Landscape, care, and the relational self: therapeutic encounters in rural England. Health Place. (2005) 11:337–48. 10.1016/j.healthplace.2005.02.004 15886142

[71] Twigger-Ross C Bonaiuto M Breakwell G. (2003). Identity Theories and Environmental Psychology. New York, NY: Routledge.

[72] Waterman AS. Personal expressiveness: philosophical and psychological foundations. J Mind Behav. (1990) 11:47–73.17222899

[73] Bonaiuto M Aiello A Perugini M Bonnes M Ercolani AP. Multidimensional perception of residential environment quality and neighbourhood attachment in the urban environment. J Environ Psychol. (1999) 19:331–52. 10.1006/jevp.1999.0138

[74] Kyle G Graefe A Manning R Bacon J. Predictors of behavioral loyalty among hikers along the Appalachian Trail. Leisure Sci. (2004) 26:99–118. 10.1080/01490400490272675

[75] Bonaiuto M Bonnes M Continisio M. Neighborhood evaluation within a multiplace perspective on urban activities. Environ Behav. (2004) 36:41–69. 10.1177/0013916503251444

[76] Mao Y Luo X Guo S Xie M Zhou J Huang R. Validation of the abbreviated indicators of perceived residential environment quality and neighborhood attachment in China. Front Public Health. (2022) 10:925651–925651. 10.3389/fpubh.2022.925651 35983368 PMC9378985

[77] Freire T. Leisure experience and positive identity development in adolescents. In: Positive Leisure Science. Berlin: Springer (2013). p. 61–79.

[78] Heo J Lee Y Pedersen PM McCormick BP. Flow experience in the daily lives of older adults: An analysis of the interaction between flow, individual differences, serious leisure, location, and social context. Can J Aging. (2010) 29:411–23. 10.1017/S0714980810000395 20707938

[79] Cohen S. Searching for escape, authenticity and identity: Experiences of lifestyle travellers. Tour Leisure Exp Consumer Manag Perspect. (2010) 44:27. 10.21832/9781845411503-005

[80] Lee TH Shen YL. The influence of leisure involvement and place attachment on destination loyalty: Evidence from recreationists walking their dogs in urban parks. J Environ Psychol. (2013) 33:76–85. 10.1016/j.jenvp.2012.11.002

[81] Csikszentmihalyi M Larson R. Flow and the Foundations of Positive Psychology. Dordrecht: Springer (2014). 10, p. 978–94.

[82] Lee IS Brown G King K Shipway R. Social identity in serious sport event space. Event Manag. (2016) 20:491–9. 10.3727/152599516X14745497664352

[83] Orta A Sicilia A Fernández-Balboa JM. Relationship between flow and athletic identity: the case of three elite sportsmen. Quest. (2017) 69:187–204. 10.1080/00336297.2016.1175951

[84] Pitt H. Therapeutic experiences of community gardens: putting flow in its place. Health Place. (2014) 27:84–91. 10.1016/j.healthplace.2014.02.006 24583563

[85] Korpela KM. Place-identity as a product of environmental self-regulation. J Environ Psychol. (1989) 9:241–56. 10.1016/S0272-4944(89)80038-6

[86] Bow V Buys E. Sense of community and place attachment: the natural environment plays a vital role in developing a sense of community. In: Social Change in the 21st Century 2003 Conference Refereed Proceedings: Centre for Social Change Research, School of Humanities and Human Services. QUT: Centre for Social Change Research, School of Humanities and Human Services. (2003). p. 1–18.

[87] Kyle GT Mowen AJ Tarrant M. Linking place preferences with place meaning: An examination of the relationship between place motivation and place attachment. J Environ Psychol. (2004) 24:439–54. 10.1016/j.jenvp.2004.11.001

[88] Devine-Wright P Howes Y. Disruption to place attachment and the protection of restorative environments: a wind energy case study. J Environ Psychol. (2010) 30:271–80. 10.1016/j.jenvp.2010.01.008

[89] Vidal T Troffa R Valera S Fornara F. Place identity as a useful psychological construct for approaching modern social challenges and new people-environment relations: residential mobility, restorative environments, and landscape. Role Place Identity Percep Understand Design Built Environ. (2012) 78–91. 10.2174/978160805413811201010078

[90] Wilkie S Stavridou A. Influence of environmental preference and environment type congruence on judgments of restoration potential. Urban Forest Urban Green. (2013) 12:163–70. 10.1016/j.ufug.2013.01.004

[91] Wilkie S Clouston L. Environment preference and environment type congruence: Effects on perceived restoration potential and restoration outcomes. Urban Forest Urban Green. (2015) 14:368–76. 10.1016/j.ufug.2015.03.002

[92] Fornara F. Are “attractive” built places as restorative and emotionally positive as natural places in the urban environment. In: Urban diversities-Environmental and Social Issues. Advances in People Environment Studies, Vol. 2. Cambridge, MA: Hogrefe and Huber Publishers (2011). p. 159–69.

[93] Jarratt D. Sense of place at a British coastal resort: exploring “seasideness” in Morecambe. Tour Interdiscip J. (2015) 63:351–64.

[94] Morton TA van der Bles AM Haslam SA. Seeing our self reflected in the world around us: the role of identity in making (natural) environments restorative. J Environ Psychol. (2017) 49:65–77. 10.1016/j.jenvp.2016.11.002

[95] Bornioli A Parkhurst G Morgan PL. The psychological wellbeing benefits of place engagement during walking in urban environments: a qualitative photo-elicitation study. Health Place. (2018) 53:228–36. 10.1016/j.healthplace.2018.08.018 30195155

[96] Liu Q Fu W den Bosch V Konijnendijk CC Xiao Y Zhu Z. Do local landscape elements enhance individuals' place attachment to new environments? A cross-regional comparative study in China. Sustainability. (2018) 10:3100. 10.3390/su10093100

[97] Liu Q Wu Y Xiao Y Fu W Zhuo Z van den Bosch CCK. More meaningful, more restorative? Linking local landscape characteristics and place attachment to restorative perceptions of urban park visitors. Lands Urban Plan. (2020) 197:103763. 10.1016/j.landurbplan.2020.103763

[98] Knez I Ode Sang Å Gunnarsson B Hedblom M. Wellbeing in urban greenery: the role of naturalness and place identity. Front Psychol. (2018) 9:491. 10.3389/fpsyg.2018.00491 29695984 PMC5904257

[99] Tsaur SH Liang YW Lin WR. Conceptualization and measurement of the recreationist-environment fit. J Leisure Res. (2012) 44:110–30. 10.1080/00222216.2012.11950257

[100] Boffi M Riva E Rainisio N Inghilleri P. Social psychology of flow: a situated framework for optimal experience. In: Flow Experience. Cham: Springer (2016). p. 215–31.

[101] Houge Mackenzie S Brymer E. Conceptualizing adventurous nature sport: a positive psychology perspective. Annals Leisure Res. (2020) 23:79–91. 10.1080/11745398.2018.1483733

[102] Rosenbaum MS Massiah C. An expanded servicescape perspective. J Serv Manag. (2011) 22:471–90. 10.1108/09564231111155088

[103] Bell SL Phoenix C Lovell R Wheeler BW. Seeking everyday wellbeing: the coast as a therapeutic landscape. Soc Sci Med. (2015) 142:56–67. 10.1016/j.socscimed.2015.08.011 26284745

[104] Lee HJ Son YH Kim S Lee DK. Healing experiences of middle-aged women through an urban forest therapy program. Urban Forest Urban Green. (2019) 38:383–91. 10.1016/j.ufug.2019.01.017

[105] Giusti M Samuelsson K. The regenerative compatibility: a synergy between healthy ecosystems, environmental attitudes, and restorative experiences. PLoS ONE. (2020) 15:e0227311. 10.1371/journal.pone.0227311 31910442 PMC6946585

[106] Thompson CW. Linking landscape and health: The recurring theme. Landsc Urban Plan. (2011) 99:187–95. 10.1016/j.landurbplan.2010.10.006

[107] Fieldhouse J. The impact of an allotment group on mental health clients' health, wellbeing and social networking. Br J Occup Therapy. (2003) 66:286–96. 10.1177/030802260306600702

[108] Lottrup L Grahn P Stigsdotter UK. Workplace greenery and perceived level of stress: Benefits of access to a green outdoor environment at the workplace. Landsc Urban Plan. (2013) 110:5–11. 10.1016/j.landurbplan.2012.09.002

[109] Colley K Brown C Montarzino A. Restorative wildscapes at work: an investigation of the wellbeing benefits of greenspace at urban fringe business sites using ‘go-along' interviews. Landscape Research. (2016) 41:598–615. 10.1080/01426397.2016.1197191

[110] Colley K Brown C Montarzino A. Understanding knowledge workers' interactions with workplace greenspace: Open space use and restoration experiences at urban-fringe business sites. Environ Behav. (2017) 49:314–38. 10.1177/0013916516629194

[111] Velarde MD Fry G Tveit M. Health effects of viewing landscapes–Landscape types in environmental psychology. Urban Forestry Urban Green. (2007) 6:199–212. 10.1016/j.ufug.2007.07.001

[112] Bringslimark T Hartig T Grindal Patil G. Adaptation to windowlessness: do office workers compensate for a lack of visual access to the outdoors? Environ Behav. (2011) 43:469–87. 10.1177/0013916510368351

[113] Boubekri M Cheung IN Reid KJ Wang CH Zee PC. Impact of windows and daylight exposure on overall health and sleep quality of office workers: a case-control pilot study. J Clin Sleep Med. (2014) 10:603–11. 10.5664/jcsm.3780 24932139 PMC4031400

[114] Lottrup L Stigsdotter UK Meilby H Claudi AG. The workplace window view: a determinant of office workers' work ability and job satisfaction. Landscape Res. (2015) 40:57–75. 10.1080/01426397.2013.829806

[115] Yeom S Kim H Hong T Lee M. Determining the optimal window size of office buildings considering the workers' task performance and the building's energy consumption. Build Environ. (2020) 177:106872. 10.1016/j.buildenv.2020.106872

[116] Wang X Shi Y Zhang B Chiang Y. The influence of forest resting environments on stress using virtual reality. Int J Environ Res Public Health. (2019) 16:3263. 10.3390/ijerph16183263 31491931 PMC6765889

[117] Gilchrist K Brown C Montarzino A. Workplace settings and wellbeing: Greenspace use and views contribute to employee wellbeing at peri-urban business sites. Landsc Urban Plan. (2015) 138:32–40. 10.1016/j.landurbplan.2015.02.004

[118] Zurawik M. Moving through spaces–leisure walking and its psychosocial benefits for well-being: a narrative review. Hum Mov. (2020) 21:1–8. 10.5114/hm.2020.89908

[119] Gutnick L. A workplace design that reduces employee stress and increases employee productivity using environmentally responsible materials. Build Res Inform. (2007) 33:317–25.

[120] White MP Alcock I Grellier J Wheeler BW Hartig T Warber SL. Spending at least 120 minutes a week in nature is associated with good health and wellbeing. Sci Rep. (2019) 9:1–11. 10.1038/s41598-019-44097-3 31197192 PMC6565732

[121] Gritzka S MacIntyre TE Dörfel D Baker-Blanc JL Calogiuri G. The effects of workplace nature-based interventions on the mental health and well-being of employees: a systematic review. Front Psychiatry. (2020) 323. 10.3389/fpsyt.2020.00323 32411026 PMC7198870

[122] AL-Ayash A Kane RT Smith D Green-Armytage P. The influence of color on student emotion, heart rate, and performance in learning environments. Color Res Appl. (2016) 41:196–205. 10.1002/col.21949

[123] Zuo L Wu D Yuan Y Li H Yu L. Effect of arrangement and quantity of epipremnum aureum on work efficiency and subjective perceptions. Environ Sci Pollut Res. (2020) 27:17804–14. 10.1007/s11356-020-08078-8 32162222

[124] Elsadek M Liu B Xie J. Window view and relaxation: Viewing green space from a high-rise estate improves urban dwellers' wellbeing. Urban Forestry Urban Green. (2020) 55:126846. 10.1016/j.ufug.2020.126846

[125] Nejati A Shepley M Rodiek S Lee C Varni J. Restorative design features for hospital staff break areas: A multi-method study. HERD Health Environ Res Design J. (2016) 9:16–35. 10.1177/1937586715592632 26163571

[126] Pilotti M Klein E Golem D Piepenbrink E Kaplan K. Is viewing a nature video after work restorative? Effects on blood pressure, task performance, and long-term memory. Environ Behav. (2015) 47:947–69. 10.1177/0013916514533187

[127] Weintraub J Cassell D DePatie TP. Nudging flow through ‘SMART' goal setting to decrease stress, increase engagement, and increase performance at work. J Occup Organ Psychol. (2021) 94:230–58. 10.1111/joop.12347

[128] De Bloom J Rantanen J Tement S Kinnunen U. Longitudinal leisure activity profiles and their associations with recovery experiences and job performance. Leisure Sci. (2018) 40:151–73. 10.1080/01490400.2017.1356254

[129] Kuykendall L Boemerman L Zhu Z. The Importance of Leisure for Subjective Well-Being. Handbook of Well-Being. Salt Lake City, UT: DEF Publishers (2018).

[130] Wiese CW Kuykendall L Tay L. Get active? A meta-analysis of leisure-time physical activity and subjective well-being. J Positive Psychol. (2018) 13:57–66. 10.1080/17439760.2017.1374436

[131] Caldwell LL. Leisure and health: why is leisure therapeutic? Br J Guid Counsel. (2005) 33:7–26. 10.1080/03069880412331335939

[132] Elbe AM Barene S Strahler K Krustrup P Holtermann A. Experiencing flow in a workplace physical activity intervention for female health care workers: a longitudinal comparison between football and Zumba. Women Sport Phys Activity J. (2016) 24:70–7. 10.1123/wspaj.2015-0011

[133] Reid D. Mindfulness and flow in occupational engagement: Presence in doing. Can J Occup Therapy. (2011) 78:50–6. 10.2182/cjot.2011.78.1.7 21395198

[134] Dane E Brummel BJ. Examining workplace mindfulness and its relations to job performance and turnover intention. Hum Relat. (2014) 67:105–28. 10.1177/0018726713487753

[135] Kuo TH Ho LA. Individual difference and job performance: The relationships among personal factors, job characteristics, flow experience, and service quality. Soc Behav Personal Int J. (2010) 38:531–52. 10.2224/sbp.2010.38.4.531

[136] Yang J Zhang ZX Tsui AS. Middle manager leadership and frontline employee performance: bypass, cascading, and moderating effects. J Manag Stud. (2010) 47:654–78. 10.1111/j.1467-6486.2009.00902.x

[137] Ceja L Navarro J. Dynamic patterns of flow in the workplace: Characterizing within-individual variability using a complexity science approach. J Organ Behav. (2011) 32:627–51. 10.1002/job.747

[138] Mark G Iqbal ST Czerwinski M Johns P. Bored Mondays and focused afternoons: the rhythm of attention and online activity in the workplace. In: Proceedings of the SIGCHI Conference on Human Factors in Computing Systems. (2014). p. 3025–34.

[139] Gammon S Jarratt D. Keeping leisure in mind: The intervening role of leisure in the blue space–health nexus. In: Blue space, health and wellbeing. Abingdon: Routledge (2019). p. 38–51.

[140] Plester B Hutchison A. Fun times: the relationship between fun and workplace engagement. Empl Rel. (2016) 38:332–50. 10.1108/ER-03-2014-002725732331

[141] Martens Y. Creative workplace: instrumental and symbolic support for creativity. Facilities. (2011) 29:63–79. 10.1108/02632771111101331

[142] Suh A Cheung CM Ahuja M Wagner C. Gamification in the workplace: the central role of the aesthetic experience. J Manag Inform Syst. (2017) 34:268–305. 10.1080/07421222.2017.1297642

[143] Pálsdóttir AM Grahn P Persson D. Changes in experienced value of everyday occupations after nature-based vocational rehabilitation. Scand J Occup Ther. (2014) 21:58–68. 10.3109/11038128.2013.832794 24041155

[144] Xanthopoulou D Bakker AB Oerlemans WG Koszucka M. Need for recovery after emotional labor: Differential effects of daily deep and surface acting. J Organ Behav. (2018) 39:481–94. 10.1002/job.2245

[145] Lai PH Gudergan S Young T Lee K. Resident intention to invite friends, relatives, and acquaintances: the dynamic process of place identity as a motivator. Tour Manag. (2021) 84:104251. 10.1016/j.tourman.2020.104251

[146] Bernardo F Palma-Oliveira JM. Urban neighbourhoods and intergroup relations: the importance of place identity. J Environ Psychol. (2016) 45:239–51. 10.1016/j.jenvp.2016.01.010

[147] Cuba L Hummon DM. A place to call home: identification with dwelling, community, and region. Sociol Quart. (1993) 34:111–31. 10.1111/j.1533-8525.1993.tb00133.x

[148] Wang WW Fan CC. Migrant workers' integration in urban China: Experiences in employment, social adaptation, and self-identity. Eurasian Geography Econ. (2012) 53:731–49. 10.2747/1539-7216.53.6.731

[149] Zhang H Li X Frenkel SJ Zhang J. Human resource practices and migrant workers' turnover intentions: The roles of post-migration place identity and justice perceptions. Hum Resour Manag J. (2019) 29:254–69. 10.1111/1748-8583.12223

[150] Lewicka M. Place attachment, place identity, and place memory: restoring the forgotten city past. J Environ Psychol. (2008) 28:209–31. 10.1016/j.jenvp.2008.02.001

[151] Ujang N. Place attachment and continuity of urban place identity. Procedia-Social Behav Sci. (2012) 49:156–67. 10.1016/j.sbspro.2012.07.014

[152] Stewart WP Liebert D Larkin KW. Community identities as visions for landscape change. Landsc Urban Plan. (2004) 69:315–34. 10.1016/j.landurbplan.2003.07.005

[153] Cheng X Zhong W Li D. Urban neighborhood self-governance and community attachment: evidence from southwest China. Cities. (2021) 112:103128. 10.1016/j.cities.2021.103128

[154] Wang H Guo F Cheng Z. Discrimination in migrant workers' welfare entitlements and benefits in urban labour market: findings from a four-city study in China. Popul Space Place. (2015) 21:124–39. 10.1002/psp.1810

[155] Valera S Vidal T. Some cues for a positive environmental psychology agenda. In: Handbook of Environmental Psychology and Quality of Life Research. Berlin: Springer (2017). p. 41–63.

[156] Uzzell D Pol E Badenas D. Place identification, social cohesion, and environmental sustainability. Environ Behav. (2002) 34:26–53. 10.1177/0013916502034001003

[157] Korpela K De Bloom J Sianoja M Pasanen T Kinnunen U. Nature at home and at work: Naturally good? Links between window views, indoor plants, outdoor activities and employee well-being over one year. Landscape Urban Plan. (2017) 160:38–47. 10.1016/j.landurbplan.2016.12.005

[158] Halpern DF. How time-flexible work policies can reduce stress, improve health, and save money. Stress Health. (2005) 21:157–68. 10.1002/smi.1049

[159] Chiang FF Birtch TA Kwan HK. The moderating roles of job control and work-life balance practices on employee stress in the hotel and catering industry. Int J Hosp Manag. (2010) 29:25–32. 10.1016/j.ijhm.2009.04.005

[160] Mokaya S Gitari JW. Effects of workplace recreation on employee performance: the case of Kenya Utalii College. Int J Hum Soc Sci. (2012) 2:176–83.

[161] Kramer M Molenaar D Arena V Venditti E Meehan R Miller R. Improving employee health: evaluation of a worksite lifestyle change program to decrease risk factors for diabetes and cardiovascular disease. J Occup Environ Med. (2015) 57:284. 10.1097/JOM.0000000000000350 25742535 PMC4351781

[162] Cheng TM Hung SH Chen MT. The influence of leisure involvement on flow experience during hiking activity: using psychological commitment as a mediate variable. Asia Pacific J Tour Res. (2016) 21:1–19. 10.1080/10941665.2014.1002507

[163] Kennedy JJ Pretorius M. Integrating a portable biofeedback device into call centre environments to reduce employee stress: results from two pilot studies. J Workplace Behav Health. (2008) 23:295–307. 10.1080/15555240802243096

[164] Dravigne A Waliczek TM Lineberger R Zajicek J. The effect of live plants and window views of green spaces on employee perceptions of job satisfaction. Hort Sci. (2008) 43:183–7. 10.21273/HORTSCI.43.1.183

[165] Cole LB Coleman S Scannell L. Place attachment in green buildings: making the connections. J Environ Psychol. (2021) 74:101558. 10.1016/j.jenvp.2021.101558

[166] Pretty J Barton J Colbeck I Hine R Mourato S Mackerron G. Health Values From Ecosystems (The UK National Ecosystem Assessment Technical Report). Cambridge: UK National Ecosystem Assessment, United National Environment Programme World Conservation Monitoring Centre (UNEP-WCMC) (2011).

[167] Engemann K Pedersen CB Arge L Tsirogiannis C Mortensen PB Svenning JC. Residential green space in childhood is associated with lower risk of psychiatric disorders from adolescence into adulthood. Proc Nat Acad Sci. (2019) 116:5188–93. 10.1073/pnas.1807504116 30804178 PMC6421415

[168] Xiang Z Luo X Zheng R Jiang Q Zhu K Feng Y. Associations of greenness surrounding schools and self-reported depressive and anxiety symptoms in Chinese adolescents. J Affect Disord. (2022) 318:62–69. 10.1016/j.jad.2022.08.095 36058356

[169] Murphy LR. Managing job stress: an employee assistance/human resource management partnership. Personnel Rev. (1995) 24:41–50. 10.1108/00483489510079075

[170] Oruh ES Mordi C Dibia CH Ajonbadi HA. Exploring compassionate managerial leadership style in reducing employee stress level during COVID-19 crisis: the case of Nigeria. Empl Relat Int J. (2021) 43:1362–81. 10.1108/ER-06-2020-0302

